# Important learning points for setting up a dental-oncology service in Ireland

**DOI:** 10.1007/s11845-026-04295-1

**Published:** 2026-03-11

**Authors:** Harriet Byrne, Claire Curtin, Catherine S. Weadick, Rícheal Ní Riordáin, Seamus O’Reilly

**Affiliations:** 1 Cork University Dental School and Hospital, Wilton, Co Cork, Ireland; 2 Cancer Research @UCC, College of Medicine and Health, University College, Cork, Ireland; 3 Department of Medical Oncology, Cork University Hospital, Wilton, Cork, Ireland

## Introduction

Illness exposes weakness and magnifies vulnerabilities. In Ireland, recognition of the significant patient and societal impacts of cancer, and of poorer cancer outcomes relative to other countries led to the development of Ireland’s first National Cancer Strategy in 1996. This was subsequently to be followed by additional strategies in 2008 and 2017 and the establishment of the National Cancer Control Programme. For patients with cancer, these strategies led to the provision of structured, guideline-based care. Consequently, cancer survival nationally has improved significantly [1].

Nonetheless challenges remain. Dental disease is the most common, untreated non-communicable disease according to the World Health Organisation [2]. This trend has shown no improvement over the past 30 years and the most recent report in 2022, highlighted the prevalence of untreated dental disease in 3.5 billon people world-wide and in 75% of people living in middle income countries [3]. Ireland is no exception, with 18% of community dwelling adults over 54 years and 40% of over 75 years have no natural teeth remaining [4, 5].

The dental oncology interface represents the co-dependence of dental and oncology services for cancer patients who experience acute effects of anticancer therapy such as oral pain and mucositis but also potential long term consequences of both radiation therapy to the head and neck and bone modifying agents (BMAs) [6–8]. These treatment modalities expose patients to the risk of developing osteoradionecrosis (ORN) or medication-related osteonecrosis of the jaw (MRONJ), respectively [8].

In Ireland, head and neck cancer accounts for 5% of cancers and is the 7th most common cancer with over 800 patients diagnosed yearly [9]. More than 60% of these cases will present with advanced stage disease and experience significant treatment related morbidity [10]. Bone metastases are common in 3 of the 4 most prevalent cancers, noted in 65–90% of prostate cancer cases, 65–75% of breast cancers, 17–64% of lung cancers and 70–95% of multiple myeloma cases [6, 11, 12]. The precise number of patients on oncology-regime BMAs is not defined however nationally there were 14,495 patients on BMA regimes between 1994 and 2012 with a 108% case increase and a 51% age-standardised incidence rise noted in that timeframe [13].

There is state-derived dental care funding for some patients prior to head and neck radiation, this is not the case for any oncology patients on BMAs [14]. This results in significant self-reliance on dental care and fragmented referral pathways. As dental and medical practitioners, we have seen the benefits of structured dental assessment pathways for patients with head and neck cancer and the impact of reducing self-reliance in this cohort [7, 15]. Financial limitations are evident for BMA oncology patients who do not benefit from state-funded services and geographical limitations for patients outside of the dental hospital catchment areas in Cork and Dental, cannot avail of pre-radiation therapy dental assessments. The structure and financial support limits the capacity of service provision in Ireland. Irrespective of pre-therapeutic dental interventions, transitioning to community dental care and long term follow-up preventative strategies are lacking [14]. Dental care is an integral, modifiable factor within an oncology patient’s management and remains the most effective strategy to reduce both ORN and MRONJ in the oncology setting [7, 16–22].

**Fig. 1:**
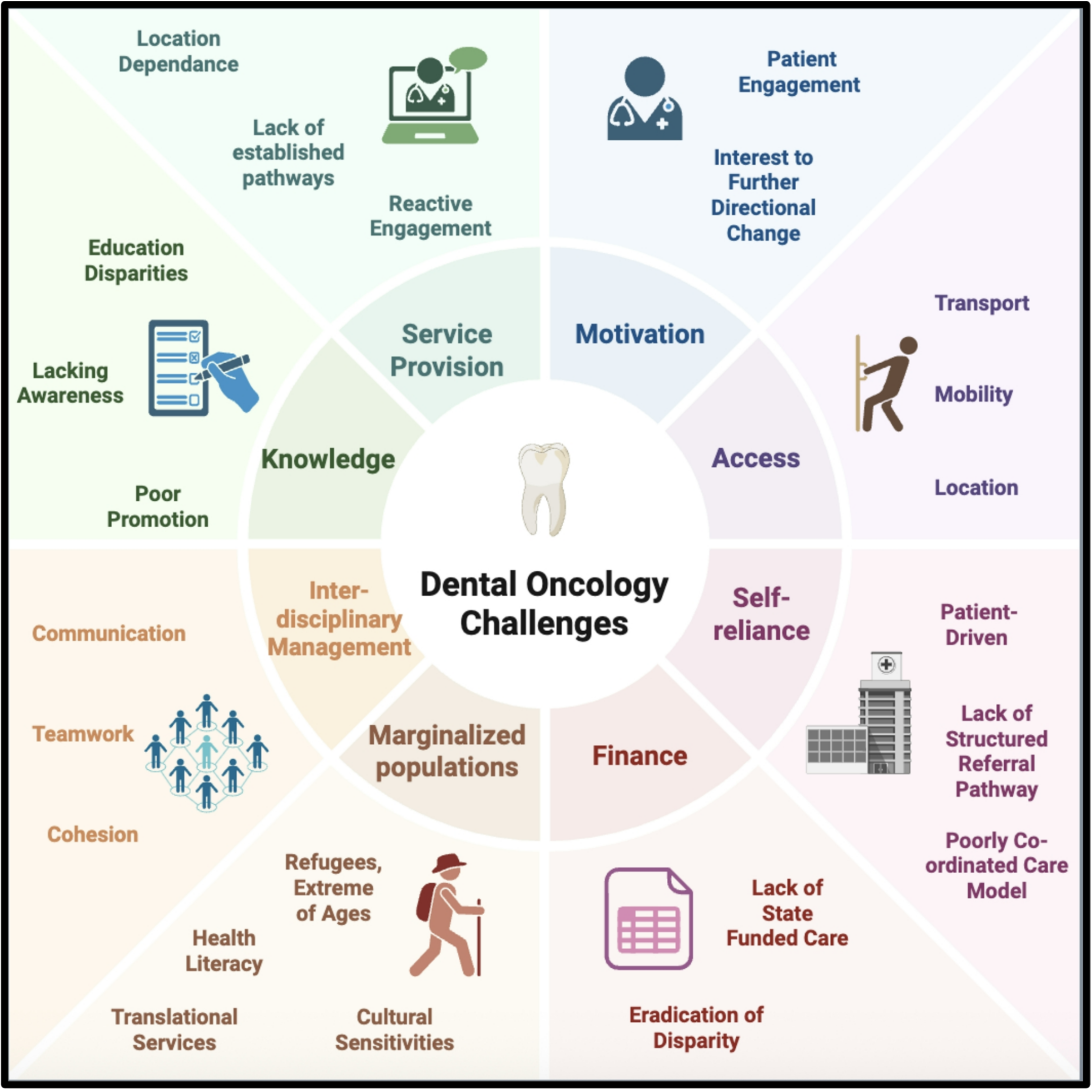
Summarises current challenges within the dental-oncology interface

We have observed a contemporaneous increase in patients on BMAs and the daily clinical challenges for patients and healthcare providers, when the dental oncology interface impacts on quality of life and treatment decision making. This prompted the establishment of a dental oncology service for these patients at Cork University Hospital/ Cork Cancer Centre which is co-located with Cork University Dental School and Hospital [21–24]. Figure 1 summarises the challenges we identified for patients and prescribers within this field. In 2026, a new cancer strategy can be developed in this paper which represents a window of opportunity and reflect on the impact of our learnings (Fig. 1). These cancer strategies could be leveraged to bridge the gap in dental-oncology and benefit patients living with cancer in our community.

### 7 learning points to setting up a dental oncology service

1. Eradication of self-reliance

Dental care remains segregated from mainstream oncology care [25]. Dental care is largely accessed by patient-driven factors and the financial burdens, timely access to care and challenges of high dental treatment needs remain challenging for patients, oncologists and dentist alike. Oral complications not only disrupt a patient’s quality of life but also impact the continuum of care. There is a perception that oral care is undervalued and the burden of care remains with patients [26]. Collaboration from the multidisciplinary team and co-ordination of preventative rather than reactive care should be prioritised [27]. Multidisciplinary team awareness and structured communication pathways across oncology and dental services reduces self-reliance for this cohort of oncology patients and streamlines access to dental care as a component of mainstream cancer care [28].

2. Education and awareness

The role of education and promotion of preventative dental strategies remains the single most effective strategy to combat the potential sequalae of anti-cancer therapy; ORN and MRONJ [7, 19, 29]. The long term effects experienced by patients with these conditions carries significant impact to both quality of life and functional capacity [30–33]. We have failed patients when cancer treatment remains a burden after treatment completion. Prescriber-led awareness is important to inform patients of possible cancer-treatment morbidities and facilitate access to dental services in this instance. Education must be provided at all levels, to empower the prescriber, patient and guide supportive services [34]. The pivotal role of education embodies holistic care and promotion of early diagnosis, and implementation of preventative and therapeutic strategies [34]. The National Comprehensive Cancer Network advocates oral health counselling prior to a following cancer therapy to account for long term treatment-related issues [35]. Educational initiatives targeting healthcare providers and cancer patients will help bridge the gap in care [34].

3. Care of vulnerable cohorts

The aging population with cancer diagnoses is growing [36]. Consequently oral health problems become more relevant, and lie outside of the established head and neck cancer screening programme [32, 36]. Ageism and identification of patients with poor dexterity, higher treatment needs, transport and cognitive deficiencies experience challenges [21, 36]. We know oral cancer and onco-therapy negatively impact a patient’s oral quality of life [26]. Young patients including survivors of certain childhood cancers lead to increased risk for poor oral health [37]. Survivors of childhood cancers present unique craniofacial and dental problems which additionally rely on the paediatric oncology teams [38]. Head and neck radiation and hematopoietic stem cell transplantation increase the risk of subsequent malignant neoplasms in the oral cavity, where survivors must be integrated into dental care for long term surveillance [37]. Risk profiling patients at the time of diagnosis may help identify urgency of care.

Refugees often face worse oral health outcomes and are disabled by challenges such as health literacy, cultural differences, institutional discrimination, and lack of financial support. A growing additional facet of dental oncology is the care of the displaced cancer patient. As of November 2025, Ireland is accommodating over 100,000 people fleeing conflict or war including over 74,000 Ukrainian refugees, and more than 21,000 international protection applicants [39]. Translation, transport and communication pose challenges for the displaced patient and consideration of social needs within oncology remains relevant [22]. Oral health tends to be poorer in refugees and migrant populations alongside poorer psychological and health related quality of life [40].

4. Interconnectivity

Dental oncology is an interdisciplinary cornerstone of patient care in the oncology setting, an aspect of cancer therapy often overlooked [34]. The burden of cancer care can often accelerate aspects such as oral care and long term dental health to deteriorate and become neglected [41]. Simple factors such as communication between medical and dental teams, patient experience, engagement with dental care, and cohesive oncology care can impact patient outcomes [42]. Communication and understanding of oncology treatment timelines, can help reduce oncology delays and reduce breaks in treatment [34, 43]. Collaboration and promotion of integrated care plans promote timely dental intervention and also establish a connect with dental services [34]. Managing the cancer patient in the primary care setting can pose challenges including the tailored training for dentists in this setting [42, 44]. There remains a deficiency of dental oncology guidelines, and resources to help manage the cancer patient challenges including treatment planning and resources to establish the correct working environment for the onco-dental team [42, 43]. This however does not lessen the importance of the primary care setting for the oncology patient. Interconnectivity and progression towards cohesion within this field should improve granularity and improve patient care [42].

5. Financial support and formalisation

Financial hardship post cancer treatment is not a new phenomena and oral complications are a common encounter during or following cancer treatment [45]. Financial toxicities has become an overwhelming burden for cancer patients adding to the weighting of cancer survivorship and quality of life [45]. In Ireland, over a 14 year period between 2009 and 2023, there was a €800 million funding for two public dental schemes. More recent times witnessed a significant drop in dental public expenditure from 63.3 million in 2017 to 39.3 million in 2021 [46]. Additional costs incurred on cancer patients in Ireland has been assessed, which included dental care as 20% of patients reported additional dental care costs. The average cost incurred was €622 as a once off payment or on average €74 per month [47]. Inconsistencies in dental care can largely be attributed to poor financial support and inadequate referral-based structure. The Irish dental system in 2025 is currently operating with a €2 million budget which is set to increase to €4 million in 2026 [48]. Efforts to mitigate disparities often lack unification and are fragmented based largely on local individual accessibility compared to nationwide driven policy [14, 49].

6. Recognition of evolving needs

Dental implants remain a controversial aspect of post cancer care particularly in head and neck cancer patients [50]. Dental implants have become a normalised, readily available option in dentistry however implant survival is greatly affected by radiation therapy alongside BMA-exposure limitations [8, 51]. There is a distinct lack of robust evidence to predict the viability of dental implants in irradiated bone and the challenges of placement in post BMA patients remains limited [50]. Specialised treatment planning collaboration between the dental and oncology plan to aim for primary implant placement with earlier prosthetic rehabilitation has shown to be more successful compared to secondary placement alongside radiation plans of less than 50 Gy [50, 52].

The viability of fixed, prosthesis for post-cancer survivors poses challenges to patients and dentists alike. The demands for the dental oncologist and specialised field becomes ever more relevant. Often patients lack a dental home, have a high treatment need and chronic inflammatory dental conditions such as periodontal disease present as a difficult condition to deem stable prior to treatment [21]. The value of an established dental home is important before and following head and neck radiation therapy and BMA therapy [8]. Establishment of secured, structured pathways to ensure preventative dental care access has been shown to satisfy not only patients but oncologists, and community dental services [53].

7. Future Proofing

Supportive cohesive cancer care, developing standardised clinical guidelines, and encouraging dental oncology training within the dental professionals can help set standards for future proofing delivery of care [41]. Currently, there is a heterogeneity in dental care support which favours locations in Dublin and Cork due to the co-location of dental hospitals. Nationwide access to dental services in both primary and secondary settings should be available for these cohorts of oncology patients. Evidence – based proformas can help optimise accurate referrals to dental centres and appropriate discharge referrals for patients returning to the primary dental care setting provide clear communication for general dental practitioners [21]. Electronic health records (HER) accessible across disciplines can help unify information instead of local storage. Allocation of a cloud-based EHR will optimise a secure, timely access to all health records ultimately reducing IT costs, improving flexibility, enhancing sharing and co-ordination of care [54, 55]. Re-integration of patients into the community for long term dental follow up also poses challenges. Telemedicine, dental oncology training, mobile health applications, dental passports and telehealth platforms particularly with limited mobility or oral concerns which can save time and early identification of any issues [34].

## Discussion

The prevalence of dental disease in Ireland is indicative of future long term oral consequences of anti-cancer therapy, particularly for patients treated with curative intent such as those with early stage breast cancer [56–58]. Two particular sequelae, ORN and MRONJ impact both quality of survival and pose significant treatment challenges [8, 30, 33, 48]. Cancer survivorship has seen improvements in recent times largely due to advances in new therapies such as targeted and immunotherapies, proactive early screening programmes and greater innovation and spending on cancer care [59]. These changes correlate with a change in onco-demographics including in Ireland where there is over a 50% increase in cancer survivors in the past decade [60]. The survival rate for head and neck cancer patients is currently 72% for 1 year and 42% for 5 years [61]. Rapidly a progressive increase in patients living with the consequences of anti-cancer therapy and dental care remains relevant to this cohort [7, 8]. Cancer incidence in Ireland has risen by 80% in the past 30 years and is expected to rise by another 30% by 2030 [62, 63]. These two aspects of oncology highlight the growing demand for dental oncology provisions internationally.

**Fig. 2:**
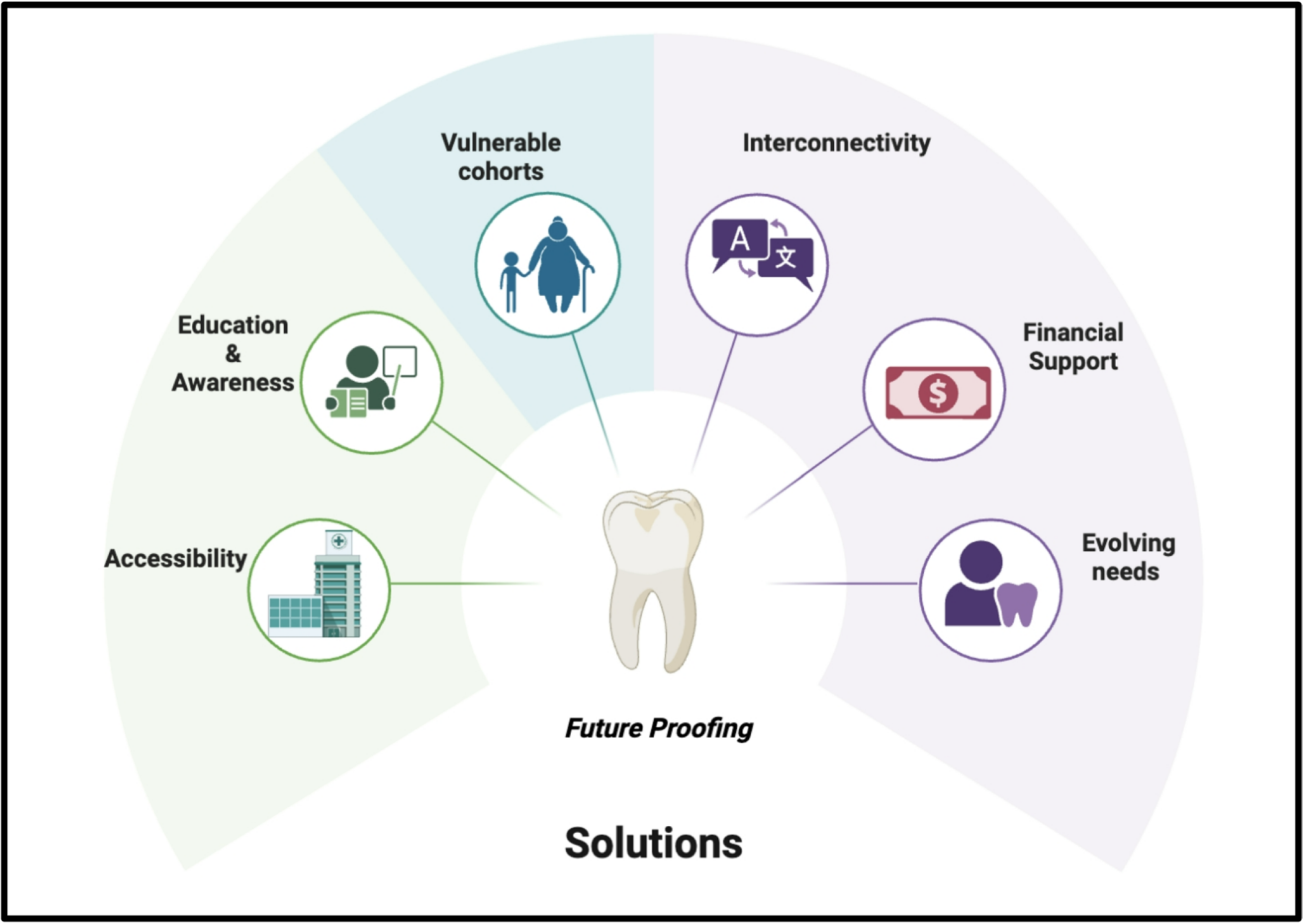
Summarises important learning points for setting up a dental oncology service in Ireland

Figure 2 summarises the themes identified in this paper to bridge the dental-oncology gap. This includes guardrails of vulnerable cohorts who remain in every aspect of cancer care, including extremes of ages and the displaced patient [21]. Dental care can particularly pose a challenge alongside everyday issues such as financial support, transport, communication, cultural sensitivities and navigation of dental systems in Ireland [60, 64–66]. Streamlining oncology care including records integration and electronic health records, formalisation of structure referral pathways and improved interconnectivity can help alleviate barriers to care [54, 67]. We understand interdisciplinary unity and cohesive care are important to patients which should be replicated in current cancer strategies [34].

Education and awareness remains the most influential tool for medical and dental specialities to improve outcomes for patients [34]. Recognition of preventative strategies and motivation to implement integrative care plans should remain integral to cancer care. Not only does proactive, preventative strategies improve patient experience and outcomes, their financial benefits remain undisputed [68]. Increasing preventative spending in the United Kingdom from 6% to 10% could lead to a £42 billion saving in the healthcare system over 10 years [68]. This is also translatable as a £13.50 return on investment for every £1 spent on prevention in the areas of maternity, oral health, diet and exercise [68]. However, systems investment often remain shortsighted and preventative strategies continue to be undervalued across the world [68]. Ireland has made attempts to promote delivery of integrated care and implement policies such as a “Healthy Ireland” to help reduce health inequalities and fragmentation [69]. Figure 1 highlights aspects of cancer care where prevention and proactivity can complement successful therapeutic outcomes can help consolidate future progression.

A key driving factor surrounding dental-oncology care is the availability of resources and an understanding of oncology patient treatment needs. Oral healthcare is isolated from mainstream oncology. As a result responsibility is placed on patients to ensure access to dental treatment and to ensure their dental needs are met. Reducing the burden of oral care and self-reliance on patients, encourages the incorporation of dental services into oncology care [70]. Optimisation of a cohesive oncodental service can incorporate timely dental needs, sufficient dental expertise, preparation of the patients for dental fitness prior to a BMA therapy or head and neck radiation therapy and coherent correspondence between GDP and oncology for long term patient care [44]. Present day cancer strategies have a lack of structured services and we would content that these new strategy can represent a drive to consolidate and expand using learning points highlighted above.

## Conclusion

Dental care remains an important element for patients exposed to head and neck radiation therapy or BMA therapy. Establishment of dental services within oncology care presents challenges to institutions, patients and clinicians alike, however addressing these needs can optimise patient care and reduce the burden of care. Understanding the core demands and requirements of an oncodental service in this article can help guide future service development.

## Supplementary Information

Below is the link to the electronic supplementary material.

ESM 1 (PDF 791 KB)

ESM 2 (DOCX 34.0 KB)
